# Early prediction of necrotizing pneumonia from mycoplasma pneumoniae pneumonia with large pulmonary lesions in children

**DOI:** 10.1038/s41598-020-76083-5

**Published:** 2020-11-04

**Authors:** Yunlian Zhou, Mengting Hu, Bei Ye, Zhimin Chen, Yuanyuan Zhang

**Affiliations:** Department of Pulmonology, Children’s Hospital, Zhejiang University School of Medicine, National Clinical Research Center for Child Health, No.3333 Binsheng Road, Hangzhou, 310052 People’s Republic of China

**Keywords:** Medical research, Signs and symptoms

## Abstract

To compare the different features of necrotizing pneumonia (NP) and non-NP (NNP) caused by Mycoplasma pneumoniae pneumonia (MPP) with large pulmonary lesions, and explore the predictor for NP to differentiate from MPP. A retrospective study of MPP patients with large pulmonary lesions hospitalized from January 2008 to December 2019 was enrolled, and clinical manifestations, laboratory findings, radiological findings were analyzed. Of 135 MPP patients with large pulmonary lesions, 56 were in the NP group, 79 were in the NNP group. We found the median length of fever days were much longer in NP group than those in NNP group. Higher levels of WBC, CRP, LDH, IL-6 in NP group were observed. Furthermore, the incidence of pulmonary consolidation was much higher in NP patients than that in NNP patients, while the CT value of large pulmonary lesion was much lower in NP patients. In ROC curve analysis, the cut-off values for the CT value and IFN-γ were 36.43 and 7.25 pg/ml, respectively. NP caused by MPP might be easier to suffer from prolonged clinical course, severe laboratory and radiological findings. CT value of large pulmonary lesions and IFN-γ could be used as biomarkers to predict NP from MPP with large pulmonary lesions in children.

## Introduction

*Mycoplasma pneumoniae* (MP) is an important cause of respiratory tract infections in children that can cause up to 20 to 40% of community-acquired pneumonia (CAP)^1^. In the past several years there have been many researchers reported that this organism can range in severity from mild to life-threatening. In general, *Mycoplasma pneumoniae* pneumonia (MPP) is self-limited, but in some cases, it can cause pulmonary and extrapulmonary complications^2^, such as necrotizing pneumonitis^3^. Necrotizing pneumonia (NP), which has been characterized by massive necrosis and liquification of lung tissues^4,5^, is commonly caused by Streptoco*ccus pneumoniae* (SP) and *Staphylococcus aureus* (SA) in children^6,7^. However, our previous study and some recent studies have reported that NP could be caused by MP^8–10^.

To the best of our knowledge, there is little literature focused on differentiating NP from MPP in children. In order to explore the predictive values of the independent related factors of NP caused by MPP with large pulmonary lesions, we retrospectively reviewed 135 children with MPP who admitted to our hospital between January 1, 2008 and December 31, 2019, then compared the differences of clinical features, laboratory data and radiological findings between NP group and non-NP (NNP) group.

## Results

### Comparison the clinical characteristics between the NP group and the NNP group

In this study, a total of 135 patients with large pulmonary lesions who were diagnosed with MPP in our hospital were enrolled. All patients had positive results of PCR test and serological detection, and had not fulfilled exclusive criteria. The median age was 5.8 (4.1–7.9) years with a female-to-male ratio of 0.82. According to the chest CT scan, patients were divided into two groups: NP group and NNP group. The clinical characteristics of these patients are presented in Table 1. There were 79 patients in the NNP group and the median age of these patients was 5.8 (4.1–7.9) years old. And there were 56 patients in the NP group, the median age was 5.1 (4.0–7.7) years old. There were no significant differences in age and gender distribution between the two groups. However, the median duration of fever and the median length of stay were significant longer in the NP group than those in the NNP group (*P* < 0.01). And the incidence of extra-pulmonary complications was 60.7% in NP group and 32.9% in NNP group, with a significant difference (*P* < 0.01).

**Table 1:** Clinical characteristic of MPP patients with large pulmonary lesions.

Clinical information	NP (n = 56)	NNP (n = 79)	*P*-value
Age, years	6.2 ± 2.5	6.2 ± 2.9	0.938
Sex (male/female)	22/34	39/40	0.294
Extra-pulmonary complications	60.7% (34/56)	32.9% (26/79)	0.002
Length of fever, days	16 (13–22)	13 (11–16)	0.001
Length of stay, days	17 (13–22)	9 (6–13)	0.000

Data are presented as mean ± SD, number (percentage), or median (25th–75th percentile).

### Comparison the laboratory characteristics between the NP group and the NNP group

These patients’ relevant laboratory data were shown in Table 2. The median levels of WBC, CRP and LDH in NP group were significantly higher than those in NNP group (P < 0.01, P < 0.05, P < 0.05, respectively). Meanwhile, we found significant lower levels of cytokines (including IL-2, IL-4, TNF-α, IFN-γ) in NP group when compared with NNP group (P < 0.01, P < 0.01, P < 0.05, P < 0.01, respectively); Patients with NP had significant higher level of IL-6 compared to that with NNP (P < 0.05). However, the median percentage of peripheral neutrophils, the median values of PCT, Immunoglobulin (including IgG, IgM, IgA), IL-10 did not differ significantly between these two groups.

**Table 2:** Laboratory characteristic of MPP patients with large pulmonary lesions.

Laboratory information	NP (n = 56)	NNP (n = 79)	*P*-value
White blood cell (× 10^9^/L)	10.49 (8.82–12.08)	7.95 (6.47–10.33)	0.001
Neutrophil, %	78.1 (67.8–82.7)	75.3 (64.8–80.5)	0.391
C-reactive protein (CRP), mg/L)	77 (37–113)	37 (17–96)	0.010
Lactatedehydrogenase (LDH), IU/L	646 (393–971)	494 (388–693)	0.030
Procalcitonin (PCT), ng/ml	0.267 (0.117–0.729)	0.195 (0.100–0.513)	0.250
**Total immunoglobulin (Ig), g/L**			
IgG	9.36 (8.00–10.88)	8.80 (6.64–11.36)	0.370
IgA	1.44 (0.78–1.80)	1.20 (0.84–1.67)	0.234
IgM	1.77 (0.91–2.80)	1.46 (1.05–2.21)	0.414
**Cytokines, pg/ml**			
Interleukin 2 (IL-2)	2.2 (1.3–3.2)	2.9 (2.3–3.8)	0.003
IL-4	2.2 (1.7–3.1)	3.1 (2.5–3.6)	0.001
IL-6	47.9 (25.9–156.5)	29.2 (13.1–76.7)	0.042
IL-10	5.7 (0.8–10.2)	5.5 (3.7–11.8)	0.386
Tumor necrosis factor alpha (TNF-α)	1.8 (0.8–2.9)	2.6 (1.5–3.6)	0.022
Interferon gamma (IFN-γ)	4.8 (0.5–13.8)	15.4 (8.1–36.4)	0.000

Data are presented as the median (25th–75th percentile), or mean ± SD.

### Comparison the radiologic characteristics between the NP group and the NNP group

Chest CT scan was performed in all patients during the hospitalization, and 56 cased of NP were identified. There were no significant differences between these two groups with respect to the incidences of pleural effusion, lobar atelectasis and pleural thickening (P ≥ 0.05) (Table 3). However, there was significant difference in the incidence of pulmonary consolidation (P < 0.01). Interestingly, we found that the mean CT value of large pulmonary lesions in NP group was significantly lower than that in NNP group (P < 0.01).

**Table 3:** Radiological features of MPP patients with large pulmonary lesions.

Radiological features	NP (n = 56)	NNP (n = 79)	*P*-value
CT Value	31.94 ± 6.84	43.52 ± 8.80	0.000
Pleural effusion	82.1% (46/56)	65.8% (52/79)	0.050
Lobar atelectasis	41.1% (23/56)	35.4% (28/79)	0.590
Pulmonary consolidation	69.6% (39/56)	29.1% (23/79)	0.000
Pleural thickening	12.5% (7/56)	10.1% (8/79)	0.783

Data are presented as mean ± SD, or number (percentage).

### Receiver operator characteristic (ROC) curves

To explore the predictors for NP caused by MPP, receiver operator characteristic (ROC) curves were made and the cut-off values with maximum sensitivities and specificities were determined. Analysis of these ROC curves showed that the CT values and IFN- γ were useful for differentiating patients with NP from MPP (Table 4). When the cut-off value for the CT values and IFN-γ were set at 36.43 and 7.25 pg/ml, respectively, the sensitivity and specificity in differentiating NP from MPP were 79.2% and 77.8%, 79.7% and 62.3%, respectively.

**Table 4:** Predictive values of the independent correlation factors in patients with NP.

Independent factors	Cutoff value	Sensitivity	Specificity	AUC	*P*-value
White blood cell (× 10^9^/L)	8.47	0.821	0.582	0.671	0.001
C-reactive protein (CRP), mg/L	43.24	0.714	0.595	0.631	0.010
Lactatedehydrogenase (LDH), IU/L	792	0.407	0.848	0.611	0.030
CT value	36.43	0.792	0.778	0.754	0.000
Interleukin 2 (IL-2), pg/ml	2.35	0.736	0.561	0.671	0.003
IL-4, pg/ml	2.35	0.819	0.581	0.694	0.001
IL-6, pg/ml	21.25	0.829	0.403	0.616	0.042
Tumor necrosis factor alpha (TNF-α)	1.85	0.684	0.519	0.617	0.022
Interferon gamma (IFN-γ)	7.25	0.797	0.623	0.703	0.000

*AUC* area under the ROC curve; *Cut-off value* the value on the ROC curve is closest to the upper right to take maximum sensitivity and specificity; *P-value* the AUC value of the independent factors compared to ROC curve reference value 0.5.

## Discussion

MPP is not only recognized as a common pathogen causing acute respiratory tract infection, such as tracheobronchitis and pneumonia, but also leads to a variety of complications^11^. NP has also been reported as a complication of MPP in which there is underlying destruction and necrosis of lung parenchyma. However, until now this is the first study focused on the different clinical characteristics of NP or NNP caused by MPP in children, and tried to elucidate the predictive factors to recognize NP early.

In the present, retrospective, observational study, 135 patients with MPP were enrolled, and the different clinical characteristics between the NP patients and NNP patients were compared. According to the diagnostic criteria of NP^4,5^, 56 cases were diagnosed as NP, while 79 were NNP. Similar age and gender distribution were observed in NP group and NNP group caused by MPP, which meant that age and gender would not be associated with the incidence of NP. In our study, we also found longer length of fever days and stay days in NP patients than those in NNP patients. Because of a longer duration of fever, prolonged hospitalization was observed in our patients, which was in accordance with the previous reports^4,12^. These results implied that NP was a refractory disease and it might be associated with significant severe destruction of pulmonary tissue.

As to laboratory data, we found significantly elevated WBC, CRP and LDH in NP patients than those in NNP patients, which were tallied with previous studies^8,13^. More interestingly, we also found the lower levels of IL-2, IL-4, TNF-α, IFN-γ, and higher level of IL-6 in NP group than those in NNP group. These results might imply the reason of host immune disorder to the development of NP. In our study, we found the obviously higher incidence of pulmonary consolidation in NP group than that in NNP group, which might due to the reason that the main radiographical evidences of NP were pulmonary consolidation before necrotic lesions appeared. Furthermore, we also found the CT value of large pulmonary lesions in NP patients was lower than that in NNP patients. This data might be related to the presence of areas of decreased parenchymal enhancement, representing liquefaction.

The diagnostic standard for NP was based on the chest CT characteristics. During the course of NP, pulmonary consolidations were firstly appeared, about 1 week later low-density lesions within the consolidation were present, then followed by multiple cavities. So, in clinics, we couldn’t diagnose NP until several weeks later the typical radiological characteristics, such as multiple small air or fluid filled cavities were shown. In order to early predict NP, using ROC curve, we analyzed some laboratory markers, which were significantly different between the two groups, to explore potential factors that may differentiate NP from NNP. Interestingly, we found that the area under the curve for CT value and IFN-γ were above 0.7 in ROC curve analysis, with the optimal cutoff value of 36.43 and 7.25 pg/ml, which indicated fair discriminative power for predicting NP caused by MPP. Furthermore, this is the first report demonstrated that CT value of large pulmonary lesions ≤ 36.43 and IFN-γ ≤ 7.25 pg/ml might help us to early predict NP from MPP with large pulmonary lesions.

Of course, our study has several limitations. Firstly, it was a retrospective study, and therefore there might have some selection bias. Secondly, there might be co-infected with other pathogens of patients though we tried our best to perform examinations. Thirdly, the distribution of patients between these two groups was not matching, which might affect the statistic results. Therefore, a further prospective study should be done to help us better understand the predictors for recognizing NP early.

In conclusion, MPP patients with NP might be easier to suffer from persistent fever, pulmonary consolidation, result in elevated WBC, CRP, LDH and disorder of cytokines. CT value of large pulmonary lesions and IFN-γ might be used as biomarkers to predict NP from MPP patients with large pulmonary lesions in children.

## Methods

### Study population

We retrospectively collected the data of 135 MPP patients with large pulmonary lesions who admitted to Children’s hospital, Zhejiang University School of Medicine between January 1, 2008 and December 31, 2019. The diagnosis of MPP was based on the signs and symptoms indicative of pneumonia^14^, as well as the positive results for serologic test (MP IgM positive and antibody titer ≥ 1:160) while having the positive results for MP polymerase chain reaction (PCR) tests of nasopharyngeal secretions. The large pulmonary lesion means the extent of infiltration on chest radiography was more than 1/3 of the lung^15^. Meanwhile, patients co-infected with other pathogens were excluded by performing some examinations, such as cultures, virus antigens detection, serology for Chlamydia pneumoniae and Legionella pneumophila, and protein purified derivative (PPD). We also excluded patients with congenital pulmonary disease, chronic cardiac and pulmonary disease, rheumatic diseases, immunodeficiency and incomplete medical records. The diagnostic standard for NP was based on the chest computed tomographic (CT) characteristics, including the loss of normal pulmonary parenchymal architecture and the presence of areas of decreased parenchymal enhancement, representing liquefaction, that are progressively replaced by multiple small air or fluid filled cavities^4,5,16^.

### Approvals

The study was approved by the ethics committee of the Children’s Hospital, Zhejiang University School of Medicine. Informed consent was obtained from at least one guardian of each patient and all procedures were conducted according to the guidelines.

### Data collection

We retrospectively collected the demographic, clinical information, laboratory data and radiological findings of patients. The samples for pathogen detection from patients were collected on admission. And blood samples were also collected on admission for the blood culture, white blood count (WBC), C-reactive protein (CRP), lactate dehydrogenase (LDH), immunoglobulins, and cytokines including interleukin (IL)-2, IL-4, IL-6, IL-10, tumor necrosis factor alpha (TNF-α) and interferon gamma (IFN-γ). According to clinical situation, all patients underwent at least one time of chest CT scan before admission or during hospitalization, and the first CT scan was performed once the chest radiograph showed large lesions. During hospitalization, body temperature, clinical signs and symptoms of all patients were followed. A febrile day means the body temperature exceeded 38.0℃ at least once during the day^17^. Extra-pulmonary complications of patients were evaluated according to our previous study^18^. And the proposal for oxygen therapy or mechanical ventilation was evaluated according to the Guidelines for management of community acquired pneumonia in children in China^19^.

### CT protocol and imaging analysis

CT was performed using 64-MDCT scanners (GE, America) for all patients, and the standard scanning parameters were as following: slice thickness of 0.625 mm, 1.375 pitch, 120 kV, and automated mA. The attenuation values (Hounsfield units, HU) were measured for all patients by two experienced radiologists who were blinded to the clinical and laboratory information. To assess the large pulmonary lesion HU values, a circular region of interest (ROI) was situated at the maximum lesion level. For each patient, three measurements were taken from the same level, and the mean HU values were compared^20^.

### Statistical analysis

We performed statistical analyses by using SPSS software (version 20.0). Normal distribution data were expressed as mean ± SD ($\overline{x}$ ± s), which were compared by independent-Samples T-test. Skewed distribution data were expressed as median values (25th-75th interquartile ranges), which were compared by the Mann–Whitney U-test. And categorical data were compared by Chi-squared tests. Receiver operating characteristic (ROC) curves were operated to evaluate candidate indicators with regards to the NP assessment of MPP patients with large pulmonary lesions.

## References

[1] Waites KB, Xiao L, Liu Y, Balish MF, Atkinson TP (2017). Mycoplasma pneumoniae from the respiratory tract and beyond. Clin. Microbiol. Rev..

[2] Poddighe D (2018). Extra-pulmonary diseases related to *Mycoplasma pneumoniae* in children: recent insights into the pathogenesis. Curr. Opin. Rheumatol..

[3] Wang Y, Xu D, Li S, Chen Z (2012). Mycoplasma pneumonia-associated necrotizing pneumonitis in children. Pediatr. Int..

[4] Sawicki GS, Lu FL, Valim C, Cleveland RH, Colin AA (2008). Necrotising pneumonia is an increasingly detected complication of pneumonia in children. Eur. Respir. J..

[5] Hansell DM (2008). Fleischner Society: glossary of terms for thoracic imaging. Radiology.

[6] Demirel N, Quizon A, Beltetón De Leon EL, Reiter J, Colin AA (2014). On the nature of pleural involvement in necrotizing pneumonia: a report of two cases of life-threatening late complications. Pediatr. Pulmonol..

[7] Ji Y (2019). Preventive effect of the phage VB-SavM-JYL01 on rabbit necrotizing pneumonia caused by Staphylococcus aureus. Vet. Microbiol..

[8] Wang X (2018). Necrotizing pneumonia caused by refractory Mycoplasma pneumonia pneumonia in children. World J. Pediatr..

[9] Chiu CY, Chiang LM, Chen TP (2006). Mycoplasma pneumoniae infection complicated by necrotizing pneumonitis with massive pleural effusion. Eur. J. Pediatr..

[10] Pellan M, Bastian C, Gaudelus J, Delacourt C, de Pontual L (2013). Pulmonary necrotizing cavity caused by *Mycoplasma pneumoniae* infection]. Arch Pediatr..

[11] de Groot RCA, Meyer Sauteur PM, Unger WWJ, van Rossum AMC (2017). Things that could be *Mycoplasma pneumoniae*. J Infect..

[12] Chen KC, Su YT, Lin WL, Chiu KC, Niu CK (2003). Clinical analysis of necrotizing pneumonia in children: three-year experience in a single medical center. Acta Paediatr. Taiwan..

[13] Hacimustafaoglu M, Celebi S, Sarimehmet H, Gurpinar A, Ercan I (2006). Necrotizing pneumonia in children. J. Trop. Pediatr..

[14] Nelson S (1998). A randomized controlled trial of filgrastim as an adjunct to antibiotics for treatment of hospitalized patients with community-acquired pneumonia, CAP study group. J. Infect. Dis..

[15] Suzuko U (2011). Japanese guidelines for the management of respiratory infectious diseases in children 2007 with focus on pneumonia. Pediatr. Int..

[16] Ramgopal S, Ivan Y, Medsinge A, Saladino RA (2017). Pediatric necrotizing pneumonia: a case report and review of the literature. Pediatr. Emerg. Care..

[17] Suzuki S (2006). Clinical evaluation of macrolide-resistant *Mycoplasma pneumoniae*. Antimicrob. Agents Chemother..

[18] Zhou Y (2014). More complications occur in macrolide-resistant than in macrolide-sensitive *Mycoplasma pneumoniae* pneumonia. Antimicrob. Agents Chemother..

[19] The Subspecialty Group of Respiratory Disease, The Society of Pediatrics, Chinese Medical Association, The Editorial Board, Chinese Journal of Pediatrics. Guidelines for management of community acquired pneumonia in children (the revised edition of 2013). *Chin. J. Pediatr.* **51**, 745–749 (2013). 24406226

[20] Abramowitz Y, Simanovsky N, Goldstein MS, Hiller N (2009). Pleural effusion: characterization with CT attenuation values and CT appearance. AJR Am. J. Roentgenol..

